# The incidence and risk factors for femoral head necrosis after femoral neck fracture in pediatric patients: a systematic review and meta-analysis

**DOI:** 10.1186/s13018-023-03502-4

**Published:** 2023-01-09

**Authors:** Pengfei Xin, Ziqi Li, Shaoqiang Pei, Qi Shi, Lianbo Xiao

**Affiliations:** 1 Shanghai University of Traditional Chinese Medicine, Shanghai, China; 2 The Third Affiliated Hospital, Guangzhou University of Chinese Medicine, Guangzhou, China; 3 Arthritis Research Institute of Integrated Traditional Chinese and Western Medicine, Shanghai Academy of Traditional Chinese Medicine, Shanghai University of Traditional Chinese Medicine, Shanghai, China; 4 Department of Orthopedics, Guanghua Hospital Affiliated to Shanghai University of Traditional Chinese Medicine, Shanghai, 20000 China

**Keywords:** Femoral head necrosis, Femoral neck fracture, Pediatric, Incidence, Risk factors, Meta-analysis

## Abstract

**Background:**

The incidence of avascular necrosis (AVN) after pediatric femoral neck fracture (PFNF) in the literature varies widely, and the risk factors associated with AVN after PFNF are controversial. Therefore, this study aimed to accurately investigate the incidence of AVN after PFNF and systematically evaluate and meta-classify their risk factors.

**Methods:**

A comprehensive search was performed of PubMed, Web of Science, and Embase. The pooled rate and 95% confidence interval (CI) were used to assess the incidence of AVN after PFNF, and pooled odds ratio (OR) were calculated to measure the effect sizes. In addition, we performed subgroup, stratified, and publication bias analyses.

**Results:**

A total of 30 articles were included in our meta-analysis, with 303 AVN cases among 1185 patients. The pooled incidence of AVN after PFNF was 22% (95% CI 18%, 27%). Subgroup analyses indicated Delbet type I–IV fracture incidences with AVN of 45%, 32%, 17%, and 12%, respectively. The incidence of AVN after PFNF in Asia was 19%, lower than in Africa at 36%, Europe at 26%, and North America at 23%. In addition, the larger sample size group and the earlier published literature group showed a higher incidence of necrosis. Stratified analyses showed that patient age and Delbet fracture classification were both important factors affecting AVN after PFNF (OR = 1.61, *p* = 0.02 and OR = 3.02, *p* < 0.001, respectively), while the time to treatment was not (OR = 0.9, *p* = 0.71).

**Conclusion:**

The pooled incidence of AVN after PFNF was ~ 22%; furthermore, the available evidence demonstrates that patient age and Delbet type of fracture were important influencing factors of AVN after PFNF.

**Supplementary Information:**

The online version contains supplementary material available at 10.1186/s13018-023-03502-4.

## Introduction

Pediatric femoral neck fractures (PFNFs) account for less than 1% of children’s fractures, but they are often accompanied by multiple complications, with the most common postoperative complication being avascular necrosis (AVN) [1–3]. Because of the high incidence of postoperative AVN, the controversies regarding the most appropriate management of AVN, and its consequent significant disability, it is extremely important to achieve a proper understanding of PFNF and the risk factors affecting AVN in order to minimize complications as much as possible [4–8]. According to the available literature, the incidence of AVN after PFNF varies from 0 to 53% [5, 9, 10]. A meta-analysis published by Yeranosian et al. [11] calculated the rate of AVN after PFNF to be 23% by calculating the population averaging. There are great differences in the reported incidence rates of AVN after PFNF, making it difficult for clinicians to accurately assess the risk.

There are likewise many controversies about the risk factors for the development of AVN after PFNF, including the patient’s age, the type of fracture, the time to treatment, and the methods of treatment utilized [10, 12–14]. In 2019, a multicenter study of 239 cases by Wang et al. found that age (older than 12 years old) was an independent risk factor for AVN after fracture, while the difference in Delbet type of fractures was not statistically significant [10]. However, multiple previous studies have shown that patient age is not a risk factor for post fracture necrosis [14–16], and AlKhatib et al. [17] conducted a meta-analysis of 6 studies and reported that Delbet type correlates with AVN after PFNF. The impact of the time from injury to treatment on the incidence of AVN after PFNF is also highly controversial. Yeranosian et al. reported that a delay in treatment of over 24 h was associated with a higher incidence of AVN [11]. AlKhatib’s report, on the other hand, came to the opposite conclusion [17]. The small sample size of the reported clinical studies and the complexity of complication-related risk factors are factors that have led authors different conclusions on the risk factors associated with AVN after PFNF.

Therefore, this study aimed to systematically review the literature to quantify the incidence of AVN after PFNF through a comprehensive search, and to explore the reasons for the wide variation in reported rates of AVN through subgroup analysis. Second, we wanted to explore the risk factors in this updated meta-analysis and to assess the effects of age, Delbet fracture type, and time to treatment on the rate of AVN in adolescent patients with femoral neck fracture.

## Methods

### Search strategies and selection criteria

The meta-analysis was designed and performed in accordance with the MOOSE (Meta-analysis of Observational Studies in Epidemiology) and the Preferred Reporting Items for Systematic Reviews and Meta-Analysis (PRISMA) guidelines. [18, 19] We searched the electronic databases of PubMed, Embase, and Web of Science up to June 2022 using the terms “adolescent,” “teen,” “pediatric,” “femur neck fracture,” “femoral neck fracture,” “avascular necrosis of the femoral head,” “ischemic necrosis of the femoral head,” and “femoral head necrosis.” (See additional file 1) The search was limited to the English language literature.

Eligible studies were selected based on the following criteria: (1) age less than 18 years; (2) femoral neck fracture classification was Delbet type; and (3) Delbet fracture type, age, or time from injury to treatment in patients with AVN were described in detail. The exclusion criteria were as follows: (1) repeatedly published data or studies; (2) single case reports and reviews; and (3) studies that included only one type of fracture. Any dispute was resolved by discussion.

### Data extraction and quality assessment

The two authors (P-FX and Z-QL) independently evaluated the retrieved articles by reading the title and abstract and evaluated all articles that met the criteria by obtaining the full text. The following information was collected: first author, year of publication, country, the total number of patients, age of patients, time to follow-up, Delbet type of fracture, number of patients with AVN, and time from injury to treatment. The Delbet classification includes four types: type I (transepiphyseal), II (transcervical), III (cervicotrochanteric), and IV (intertrochanteric) [20]. We define them as two groups (Delbet I-II type versus Delbet III-IV type). Patients were defined as having had early treatment (≤ 24 h) and late treatment (> 24 h) according to the time from injury to treatment. Low age (≤ 12 years) and older age (> 12 years) groups were defined according to age. The methodological study quality assessment of the included case–control or retrospective cohort studies used the modified Newcastle–Ottawa scale (NOS), and the case series studies were assessed using a checklist that was developed at the Institute of Health Economics (IHE) [21, 22]. Study quality was evaluated independently by two reviewers.

### Statistical analysis

We performed the meta-analysis using Stata 12.0 software and RevMan 5.2 software. Data were extracted, and the pooled incidence rate of AVN after PFNF and the 95% confidence intervals (CIs) were calculated. In cases where a study reported no events of AVN, we applied a continuity correction of 0.5 to each cell to allow for analysis [23]. Subgroup analysis was performed according to the year of publication, country, sample size, Delbet type, and patient age. Moreover, stratified analyses were performed to evaluate the risk of AVN after PFNF in patients with Delbet I–II versus Delbet III–IV fracture types, patients with low age versus older age groups, and patients with early versus late treatment. An odds ratio (OR) with 95% confidence interval (CI) was summarized. For heterogeneity analysis, a *Q* test (*P* > 0.10) or *I*^2^ test < 50% was considered homogeneity among studies, and the fixed effect model was adopted; in contrast, when heterogeneity among studies was detected, a random-effects model was used. Sensitivity analysis was conducted by a study removal approach to evaluate the quality and consistency of the results. Publication bias was assessed by funnel plots and Egger’s test [24].

## Results

### Details regarding the included literature

**Fig. 1:**
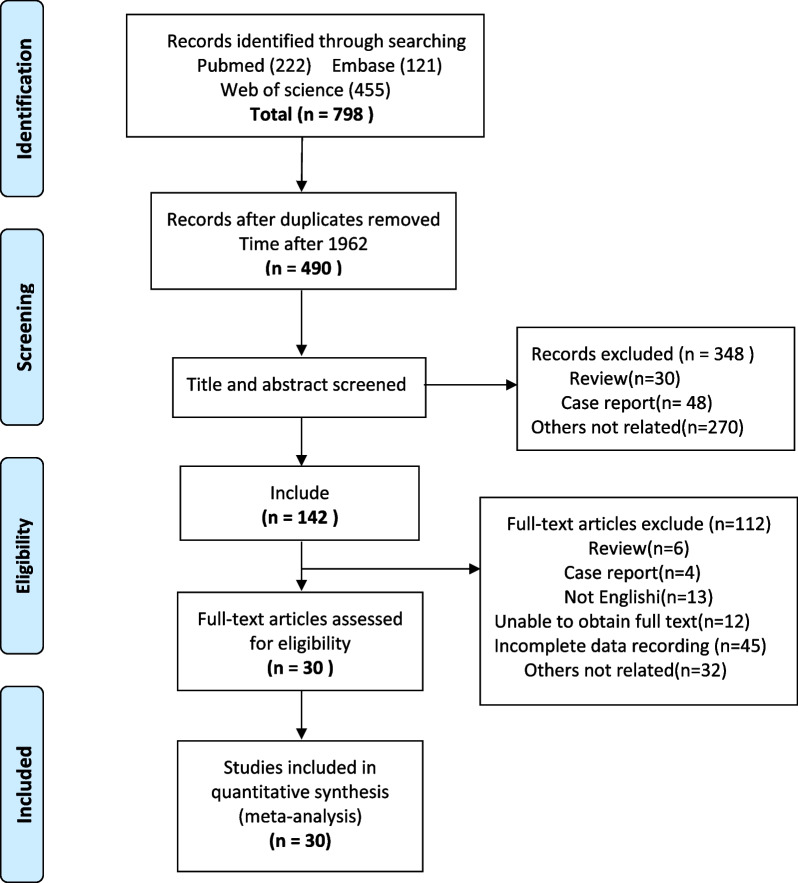
PRISMA flow diagram

A total of 798 articles were identified from the three databases, and a total of 380 articles were removed due to article repetition. After reading the title, abstract, and full text, we determined that 30 articles met the inclusion criteria for our meta-analysis. The flowchart depicting the PRISMA decision process can be seen in Fig. 1. In this study, studies were published between 1962 and 2022 and performed in China [10, 25–31], America [2, 9, 12, 14, 32–35], India [15, 36, 37], Turkey [5, 16, 38], Serbia [39], Austria [40], Morocco [41], Saudi Arabia [42], Egypt [43], Germany [44], Canada [45], and England [20]. Eleven were retrospective case–control studies, one was a retrospective cohort study, and eighteen were case series studies. Detailed information on the 30 included articles is shown in Table 1.

**Table 1:** Characteristics of the included studies on the femoral head necrosis after femoral neck fracture

Authors	Country	Year	Study design	Patients/fractures (n)	Mean age (years) (range)	Number of AVN	Incidence rate (%)	NOS/IHE score
Li [25]	China	2022	Case–control	28	9.4 (6.2–12.1)	6	21.4	7^a^
Wu [26]	China	2020	Case–control	16/17	10.4 (1–14)	4	23.53	7^a^
Singh [36]	India	2020	Case–control	32/34	11.7 (4–17)	4	11.76	8^a^
Dai [27]	China	2020	Case–control	44	9 (2–14)	14	31.82	8^a^
Wang [10]	China	2019	Case–control	239/241	10	59	24.48	8^a^
Spence [12]	USA	2016	Case–control	70	13.2 (1.3–18.1)	20	28.57	7^a^
Liu [28]	China	2016	Case series	21	15.3 (14–18)	1	4.76	13^b^
Stone [32]	USA	2015	Case–control	22	11.5 (4.5–17.4)	8	36.36	7^a^
Patrick [33]	USA	2015	Case–control	43/44	0.9–17	9	20.45	6^a^
Bukva [39]	Serbia	2015	Case–control	28	10.7 (4–14)	11	39.29	6^a^
Hajdu [40]	Austria	2011	Cross-sectional	8	11.6 (3–15)	1	12.50	9^a^
Bali [37]	India	2011	Case series	36	10 (3–16)	7	19.44	11^b^
Dendane [41]	Morocco	2010	Case series	21	12.1 (5–16)	7	33.33	13^b^
Inan [16]	Turkey	2009	Case–control	39	11.1 (4–16)	11	28.21	8^a^
Varshney [15]	India	2009	Case–control	21	11.8 (5–15)	3	14.29	8^a^
Qi [29]	China	2008	Case series	23	10.2 (5–16)	3	13.04	11^b^
Shrader [14]	USA	2007	Case series	20	11 (4–15)	2	10.00	15^b^
Togrul [38]	Turkey	2005	Cross-sectional	61/62	10.2 (2–14)	9	14.52	10^b^
Mirdad [42]	Saudi Arabia	2002	Case series	14	9.1 (4–16)	7	50.00	9^b^
Flynn [34]	USA	2002	Case series	18	8 (2–13)	1	5.56	11^b^
Bagatur [5]	Turkey	2002	Case series	17	11 (7–14)	9	52.94	9^b^
Song [9]	USA	2001	Case series	13	5–16	0	0.00	10^b^
Morsy [43]	Egypt	2001	Case series	53	10.2 (3–16)	21	39.62	9^b^
Pape [44]	Germany	1999	Case series	28	11.8 (1–15)	3	10.71	12^b^
Cheng [30]	China	1999	Case series	14	3–16	0	0.00	12^b^
Ng [45]	Canada	1996	Retrospective cohort	32	9.5 ± 5	9	28.13	8^a^
Davison [2]	USA	1992	Case series	19	11 (1–16)	9	47.37	8^b^
Canale [35]	USA	1977	Case series	60/61	9.7 (0.5–17)	26	42.62	8^b^
Lam [31]	China	1971	Case series	75	0.7–17	11	14.67	10^b^
Ratliff [20]	UK	1962	Case series	71	< 17	30	42.25	9^b^

*AVN* avascular necrosis, *NA* missing value

^a^NOS = Newcastle–Ottawa Scale (0–9 points)

^b^IHE = Institute of Health Economics(0–20 points); The higher the score, the higher the quality of the study

### Quality assessment

Methodological quality was considered high for studies with NOS scores equal to or greater than 6 [21]. Twelve studies were evaluated as having high methodological quality in accordance with the NOS. Based on the IHE tool, the checklist consists of 20 criteria, and eighteen studies were reviewed by answering “yes,” “partial,” “no,” or “unclear.” We then tallied the number of “yes” and “partial” answers to estimate the risk of bias [22]. In total, the mean study IHE score was 10.5 of 20 possible points. The scoring system of IHE does not assign a minimum score in which a certain level of quality is achieved [22]; for the case series, the average score was judged to be fair.

### The incidence of AVN after PFNF

**Fig. 2:**
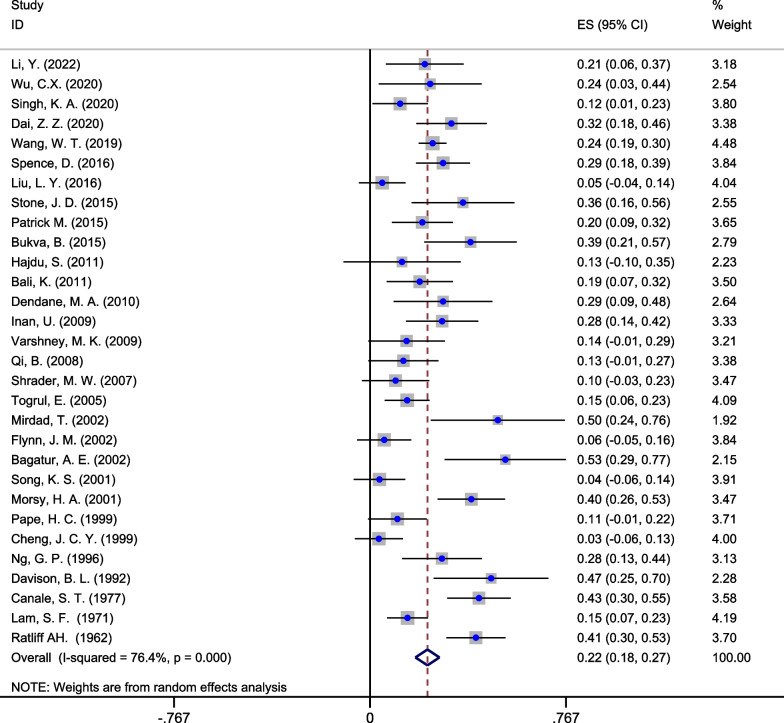
Forest plot of pooled incidence rate of AVN after PFNF

**Fig. 3:**
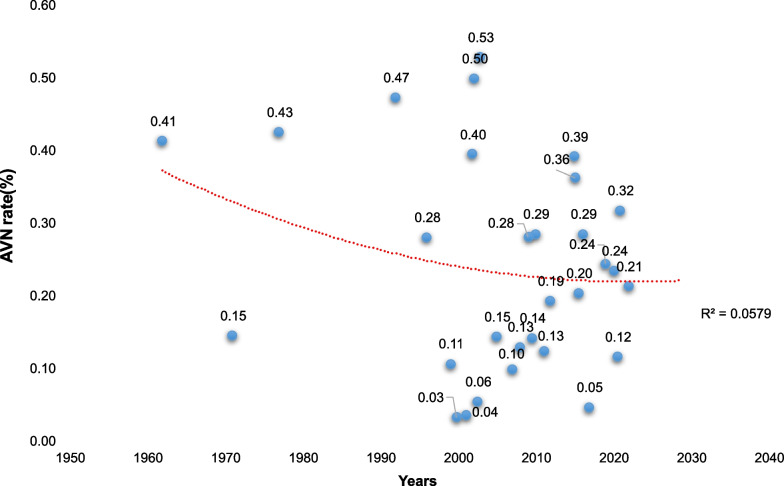
AVN incidence trended for publishing literature

A total of 1185 patients (1193 hips) were included in the study, including 303 hips with AVN after PFNF, ranging in age from 0 to 18 years. The overall pooled incidence of AVN after PFNF was 22% (95% CI 18%, 27%). As the heterogeneity test showed heterogeneity among the studies (*I*^2^ = 76.4%, *p* < 0.001), the random-effects model was used for the meta-analysis (Fig. 2). The AVN rates of all included articles are shown in Fig. 3.

Subgroup analyses showed that the pooled incidence of AVN after PFNF in 2000–2009 was 21%, lower than in 1962–1999 at 26% and in 2010–2020 at 22%. The pooled incidence of AVN after PFNF in Asia was 19%, lower than in Africa at 36%, Europe at 26%, and North America at 23%. The pooled incidence of AVN after PFNF was 45% for Type I fractures, 32% for Type II fractures, 17% for Type III fractures, and 12% for Type IV fractures (see Table 2). Subgroup analyses also showed studies with larger sample sizes than those with smaller sample sizes (28% vs. 19%, *p* < 0.001) and older age group than low age group (28% vs. 22%; Table 2).

**Table 2:** Subgroup analysis of femoral head necrosis rate

Subgroups	No. of studies	No. of total fractures	Number of AVN	AVN incidence (%)	95% CI	*I*^2^ (%)	*P* value	Model
*Time*								
1962–1999	7	299	87	26	13–39	87.8	< 0.001	R
2000–2010	11	301	72	21	13–30	76.8		
2011–2022	12	593	144	22	16–28	60.3		
*Country*								
Africa	2	74	27	36	25–47	0	< 0.001	R
Europe	4	134	44	26	8–44	82.4		
North America	9	299	84	23	13–34	81.8		
Asian	15	686	148	19	14–25	69.9		
*Sample size*								
≥ 39	10	759	209	28	22–34	73.7	< 0.001	R
< 39	20	435	95	19	13–25	69.5		
*Delbet grade*								
Delbet I	22	60	34	45	31–59	0	0.859	F
Delbet II	27	587	191	32	26–39	66.1	< 0.001	R
Delbet III	28	416	57	17	14–21	0	0.740	F
Delbet IV	23	103	5	12	2–22	0	0.987	F
*Age*								
< 12 year	13	289	61	22	14–30	50.9	0.026	R
≥ 12 year	13	281	85	28	18–39	71.6	< 0.001	R

*AVN* avascular necrosis, *F* fixed effect model, *R* random-effects model

### Risk factors for AVN after PFNF

**Fig. 4:**
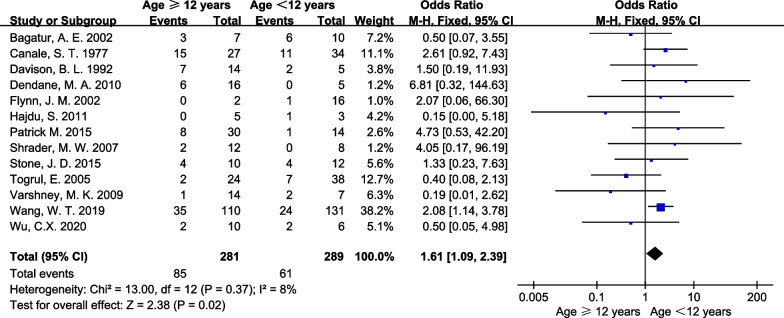
Forest plot: pooled odds ratio (OR) for AVN in the low age group (≤ 12 years) versus older age group (> 12 years) PFNF

Of the 30 included studies, 13 reported [2, 5, 8, 12, 13, 22, 28–31, 34, 36, 37] in detail the correlation between age and AVN after PFNF. Pooled results with a fixed effect model showed that older age group (> 12 years) were associated with a higher risk of AVN after PFNF (OR: 1.61, 95% CI 1.09 and 2.39, *p* = 0.02; *I*^2^ = 8%; Fig. 4).

**Fig. 5:**
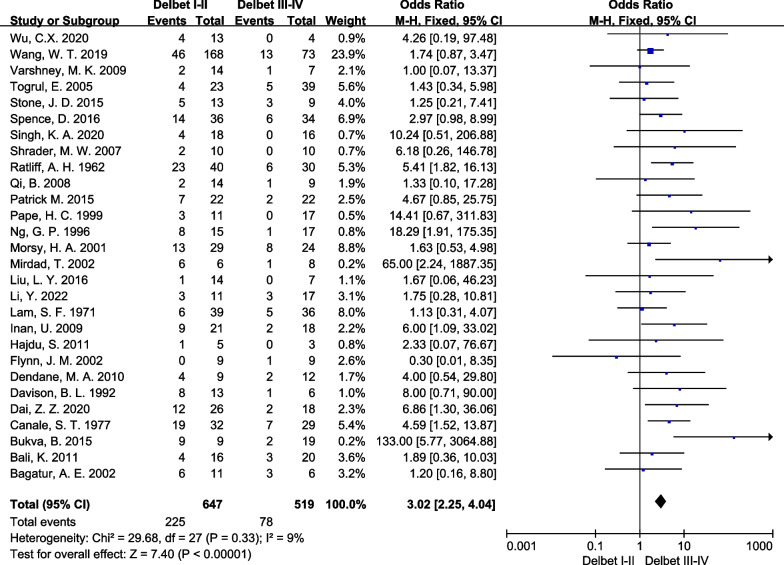
Forest plot: pooled odds ratio (OR) for AVN in the Delbet type I-II versus Delbet type III-IV PFNF

**Fig. 6:**
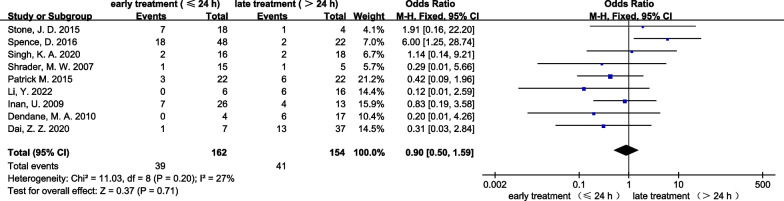
Forest plot: pooled odds ratio (OR) for AVN in the early treatment (≤ 24 h) versus late treatment (> 24 h) PFNF

Data on Delbet classification were available for the meta-analysis from twenty-eight studies, and the fixed effect model was used since there was no obvious heterogeneity (*I*^2^ = 9%, *P* < 0.001). The analysis showed that AVN was more associated with Delbet type I-II fractures in comparison to Delbet type III–IV fractures (OR: 3.02, 95% CI 2.25 and 4.04, *p* < 0.001; Fig. 5). However, comparison of the comparative effect of early (≤ 24 h) versus late (> 24 h) treatment of PFNF showed no significant difference in the rate of AVN (OR: 0.9, 95% CI 0.50 and 1.59, *p* = 0.71; Fig. 6).

### Sensitivity analyses and publication bias

**Fig. 7:**
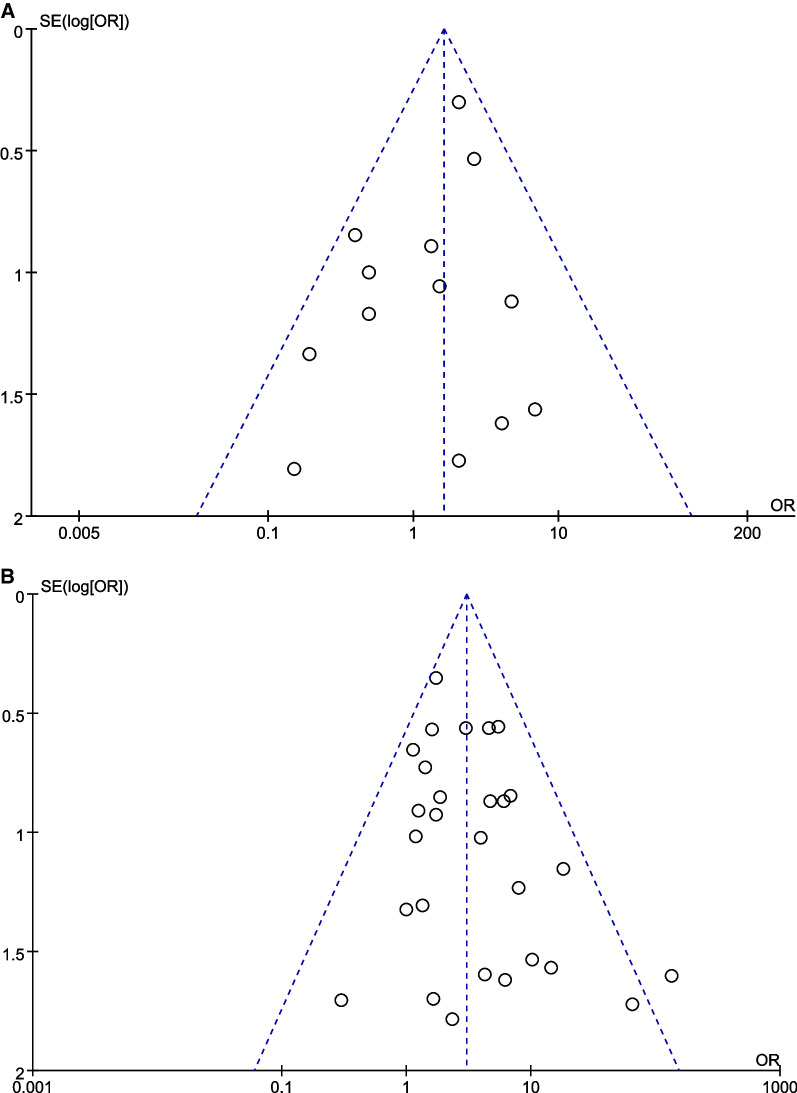
Study publication bias funnel plot; **a** patient age profile published bias funnel, and **b** Delbet classification situation published bias funnel

Sensitivity analysis was performed on the selected studies to assess whether any individual studies affected the total femoral head necrosis rate, and the outcomes suggested relatively good agreement among studies. (See additional file 2) The funnel plots of patient age and Delbet fracture type publication bias are shown in Fig. 7a, b. Visual inspection indicates that these plots are symmetrical, suggesting low risk of publication bias. Egger’s regression test also indicated a low probability of publication bias (*p* = 0.240 and 0.121, respectively).

## Discussion

In this meta-analysis, we found that the overall incidence of AVN after PFNF was 22% in the 30 studies that were included, which was comparable to the two previously reported studies with large sample sizes [10, 11]. Subgroup analyses showed that the year of publication, geographic locale of the populations studies, and sample size of the literature may be reasons for the large variations seen in the incidence of AVN. In addition, stratified analyses showed that the Delbet fracture type and the age of patients were critical risk factors for AVN after PFNF, while the time to treatment was not. These findings may help inform treatment decisions after adolescent femoral neck fractures.

In our study, we found that adolescents older than 12 years were 1.61 times more likely to develop AVN than adolescents younger than 12 years (*P* = 0.02). Several clinical studies in recent years have also confirmed our findings [10, 25]. The reasons for this difference are mostly believed to be related to the vascular distribution of the proximal femur in children of different ages and the reconstructing ability of children [25, 46, 47]. We believe that the reason for this discrepancy may be related to the fact that the older the age group, the more active the adolescent is and thus more vulnerable to greater violence of injury.

Whether the Delbet fracture type is correlated with the incidence of AVN has been a controversial topic among surgeons. Reporting on the long-term follow-up of 61 children, Emre et al. [38] proposed that the severity of the initial trauma of the fracture is closely related to the incidence of AVN. Moon et al. [48], in their meta-analysis, found fracture type to be the most important predictor of AVN. Ulukan et al. [16] conducted a retrospective study of 39 patients and found that the type of reduction was not significantly related to the incidence of AVN, although the type of fracture was correlated with AVN occurrence, as was also reported by several subsequent studies [12, 17]. In our study, Delbet type of fractures was also found to be an important risk factor for AVN after PFNF, and the pooled necrosis rate of Delbet type I-II fractures was as high as 45% and 32%, respectively. Therefore, vigilance is particularly needed when we encounter these two types of fractures in clinical practice.

This study has limitations that should be noted. First, a large degree of heterogeneity was detected between different studies, which may be related to small sample sizes, differing inclusion criteria, large time span, variability in treatment, and other differences, resulting in variation in results between the studies. Second, in our study, the subgroup analysis included a relatively small number of studies and had limited power to make accurate conclusions. We believe that the occurrence of necrosis after fracture may depend more on the injury itself; therefore, we have not studied some risk factors that occur after injury. Third, the factors reported in the studies were not always consistent, and some studies did not adjust for confounders, which may have influenced the pooled results.

## Conclusions

In summary, our meta-analysis showed that the pooled incidence of AVN after PFNF was ~ 22% and suggests that patient age and Delbet fracture classification are important factors affecting AVN after PFNF; conversely, the evidence at present does not indicate an association between the time to treatment and the rate of AVN in adolescents. However, our study is based on the results obtained from previously reported observational studies and needs to be confirmed by large-sample multicenter studies.

## Supplementary Information

**Additional file 1:** Search strategy.

**Additional file 2:** Sensitivity analysis.

## Data Availability

The authors declare that all the data supporting the findings of this study are available within the article and its supplementary information files.

## Abbreviations

AVN: Avascular necrosis
PFNF: Pediatric femoral neck fracture
OR: Odds ratio
CI: Confidence interval
IHE: Institute of Health Economics
MOOSE: Meta-analysis of Observational Studies in Epidemiology
PRISMA: Preferred Reporting Items for Systematic Reviews and Meta-Analysis
