# Supplementary material for: The incidence and risk factors for femoral head necrosis after femoral neck fracture in pediatric patients: a systematic review and meta-analysis
Source: J Orthop Surg Res. 2023 Jan 9;18:22. doi: 10.1186/s13018-023-03502-4 (PMC9830722; doi:10.1186/s13018-023-03502-4)
Supplement: Supplementary file 1 — Additional file 1: Search strategy. [file 13018_2023_3502_MOESM1_ESM.docx]

**Web of science**

#1

TS=（child* or Pediatrics or adolescen* or Teen* or Youth* or Adolescents, Female or Adolescent, Female or Female Adolescents or Adolescents, Male or Adolescent, Male or Male Adolescent or Male Adolescents）

#2

TS=（femoral neck fracture or Femoral Neck Fracture or Femur Neck Fractures or Femur Neck Fracture ）

#3

TS=（ Femur Head Necrosis or Femur Head Necroses or Head Necrosis, Femur or Necrosis, Femur Head or Aseptic Necrosis of Femur Head or Necrosis, Aseptic, of Femur Head or Ischemic Necrosis Of Femoral Head or Femoral Head, Avascular Necrosis Of or Avascular Necrosis Of Femoral Head, Primary or Avascular Necrosis of Femur Head ）

#4：#1 and #2 and #3

**Pubmed**

#1

"Child"[Mesh]

#2

children[Title/Abstract]

#3

(children[Title/Abstract]) OR "Child"[Mesh]

#4

"Adolescent"[Mesh]

#5

(((((((((((((((Adolescents[Title/Abstract]) OR Adolescence[Title/Abstract]) OR Teens[Title/Abstract]) OR Teen[Title/Abstract]) OR Teenagers[Title/Abstract]) OR Teenager[Title/Abstract]) OR Youth[Title/Abstract]) OR Youths[Title/Abstract]) OR Adolescents, Female[Title/Abstract]) OR Adolescent, Female[Title/Abstract]) OR Female Adolescent[Title/Abstract]) OR Female Adolescents[Title/Abstract]) OR Adolescents, Male[Title/Abstract]) OR Adolescent, Male[Title/Abstract]) OR Male Adolescent[Title/Abstract]) OR Male Adolescents[Title/Abstract]

#6

(((((((((((((((((Adolescents[Title/Abstract]) OR Adolescence[Title/Abstract]) OR Teens[Title/Abstract]) OR Teen[Title/Abstract]) OR Teenagers[Title/Abstract]) OR Teenager[Title/Abstract]) OR Youth[Title/Abstract]) OR Youths[Title/Abstract]) OR Adolescents, Female[Title/Abstract]) OR Adolescent, Female[Title/Abstract]) OR Female Adolescent[Title/Abstract]) OR Female Adolescents[Title/Abstract]) OR Adolescents, Male[Title/Abstract]) OR Adolescent, Male[Title/Abstract]) OR Male Adolescent[Title/Abstract]) OR Male Adolescents[Title/Abstract])) OR "Adolescent"[Mesh]

#7

(((((((((((((((((((Adolescents[Title/Abstract]) OR Adolescence[Title/Abstract]) OR Teens[Title/Abstract]) OR Teen[Title/Abstract]) OR Teenagers[Title/Abstract]) OR Teenager[Title/Abstract]) OR Youth[Title/Abstract]) OR Youths[Title/Abstract]) OR Adolescents, Female[Title/Abstract]) OR Adolescent, Female[Title/Abstract]) OR Female Adolescent[Title/Abstract]) OR Female Adolescents[Title/Abstract]) OR Adolescents, Male[Title/Abstract]) OR Adolescent, Male[Title/Abstract]) OR Male Adolescent[Title/Abstract]) OR Male Adolescents[Title/Abstract])) OR "Adolescent"[Mesh])) OR ((children[Title/Abstract]) OR "Child"[Mesh])

#8

"Femoral Neck Fractures"[Mesh]

#9

((Femoral Neck Fracture[Title/Abstract]) OR Femur Neck Fractures[Title/Abstract]) OR Femur Neck Fracture[Title/Abstract]

#10

((((Femoral Neck Fracture[Title/Abstract]) OR Femur Neck Fractures[Title/Abstract]) OR Femur Neck Fracture[Title/Abstract])) OR "Femoral Neck Fractures"[Mesh]

#11

"Femur Head Necrosis"[Mesh]

#12

(((((((((Femur Head Necroses[Title/Abstract]) OR Head Necrosis, Femur[Title/Abstract]) OR Necrosis, Femur Head[Title/Abstract]) OR Aseptic Necrosis of Femur Head[Title/Abstract]) OR Necrosis, Aseptic, of Femur Head[Title/Abstract]) OR Necrosis, Avascular, of Femur Head[Title/Abstract]) OR Ischemic Necrosis Of Femoral Head[Title/Abstract]) OR Femoral Head, Avascular Necrosis Of[Title/Abstract]) OR Avascular Necrosis Of Femoral Head, Primary[Title/Abstract]) OR Avascular Necrosis of Femur Head[Title/Abstract]

#13

(((((((((((Femur Head Necroses[Title/Abstract]) OR Head Necrosis, Femur[Title/Abstract]) OR Necrosis, Femur Head[Title/Abstract]) OR Aseptic Necrosis of Femur Head[Title/Abstract]) OR Necrosis, Aseptic, of Femur Head[Title/Abstract]) OR Necrosis, Avascular, of Femur Head[Title/Abstract]) OR Ischemic Necrosis Of Femoral Head[Title/Abstract]) OR Femoral Head, Avascular Necrosis Of[Title/Abstract]) OR Avascular Necrosis Of Femoral Head, Primary[Title/Abstract]) OR Avascular Necrosis of Femur Head[Title/Abstract])) OR "Femur Head Necrosis"[Mesh]

#14

((((((((((((((((((((((Adolescents[Title/Abstract]) OR Adolescence[Title/Abstract]) OR Teens[Title/Abstract]) OR Teen[Title/Abstract]) OR Teenagers[Title/Abstract]) OR Teenager[Title/Abstract]) OR Youth[Title/Abstract]) OR Youths[Title/Abstract]) OR Adolescents, Female[Title/Abstract]) OR Adolescent, Female[Title/Abstract]) OR Female Adolescent[Title/Abstract]) OR Female Adolescents[Title/Abstract]) OR Adolescents, Male[Title/Abstract]) OR Adolescent, Male[Title/Abstract]) OR Male Adolescent[Title/Abstract]) OR Male Adolescents[Title/Abstract])) OR "Adolescent"[Mesh])) OR ((children[Title/Abstract]) OR "Child"[Mesh]))) AND (((((Femoral Neck Fracture[Title/Abstract]) OR Femur Neck Fractures[Title/Abstract]) OR Femur Neck Fracture[Title/Abstract])) OR "Femoral Neck Fractures"[Mesh])) AND ((((((((((((Femur Head Necroses[Title/Abstract]) OR Head Necrosis, Femur[Title/Abstract]) OR Necrosis, Femur Head[Title/Abstract]) OR Aseptic Necrosis of Femur Head[Title/Abstract]) OR Necrosis, Aseptic, of Femur Head[Title/Abstract]) OR Necrosis, Avascular, of Femur Head[Title/Abstract]) OR Ischemic Necrosis Of Femoral Head[Title/Abstract]) OR Femoral Head, Avascular Necrosis Of[Title/Abstract]) OR Avascular Necrosis Of Femoral Head, Primary[Title/Abstract]) OR Avascular Necrosis of Femur Head[Title/Abstract])) OR "Femur Head Necrosis"[Mesh])

Embase

#1 'child'/exp

#2 'children':ab,ti

#3. #1 OR #2

#4. 'adolescent'/exp

#5. 'adolescents':ab,ti

#6. 'adolescence teens':ab,ti

#7. 'adolescence':ab,ti

#8. 'teens':ab,ti

#9. 'teen':ab,ti

#10. 'teenagers':ab,ti

#11. 'teenager':ab,ti

#12. 'youth':ab,ti

#13. 'youths':ab,ti

#14. 'adolescents, female':ab,ti

#15. 'adolescent, female':ab,ti

#16. 'female adolescent':ab,ti

#17. 'female adolescents':ab,ti

#18. 'adolescents, male':ab,ti

#19. 'adolescent, male':ab,ti

#20. 'male adolescent':ab,ti

#21. 'male adolescents':ab,ti

#22. 'pediatrics':ab,ti

#23. #3 OR #4 OR #5 OR #6 OR #7 OR #8 OR #9 OR #10 OR #11 OR #12 OR #13 OR #14 OR #15 OR #16 OR #17 OR #18 OR #19 OR #20 OR #21 OR #22

#24. 'femoral neck fracture'/exp

#25. 'femoral neck fracture':ab,ti

#26. 'femur neck fractures':ab,ti

#27. 'femur neck fracture':ab,ti

#28. #24 OR #25 OR #26 OR #27

#29. 'femur head necrosis'/exp

#30. 'femur head necroses':ab,ti

#31. 'head necrosis, femur':ab,ti

#32. 'necrosis, femur head':ab,ti

#33. 'aseptic necrosis of femur head':ab,ti

#34. 'necrosis, aseptic, of femur head':ab,ti

#35. 'necrosis, avascular, of femur head':ab,ti

#36. 'ischemic necrosis of femoral head':ab,ti

#37. 'femoral head, avascular necrosis of':ab,ti

#38. 'avascular necrosis of femoral head,primary':ab,ti

#39. 'avascular necrosis of femur head':ab,ti

#40. #29 OR #30 OR #31 OR #32 OR #33 OR #34 OR #35 OR #36 OR #37 OR #38 OR #39

#41. #23 AND #28 AND #40
