# Supplementary figures and images for: The incidence and risk factors for femoral head necrosis after femoral neck fracture in pediatric patients: a systematic review and meta-analysis
Source: J Orthop Surg Res. 2023 Jan 9;18:22. doi: 10.1186/s13018-023-03502-4 (PMC9830722; doi:10.1186/s13018-023-03502-4)

# Meta-analysis estimates, given named study is omitted

| Lower CI Limit

○ Estimate

| Upper CI Limit

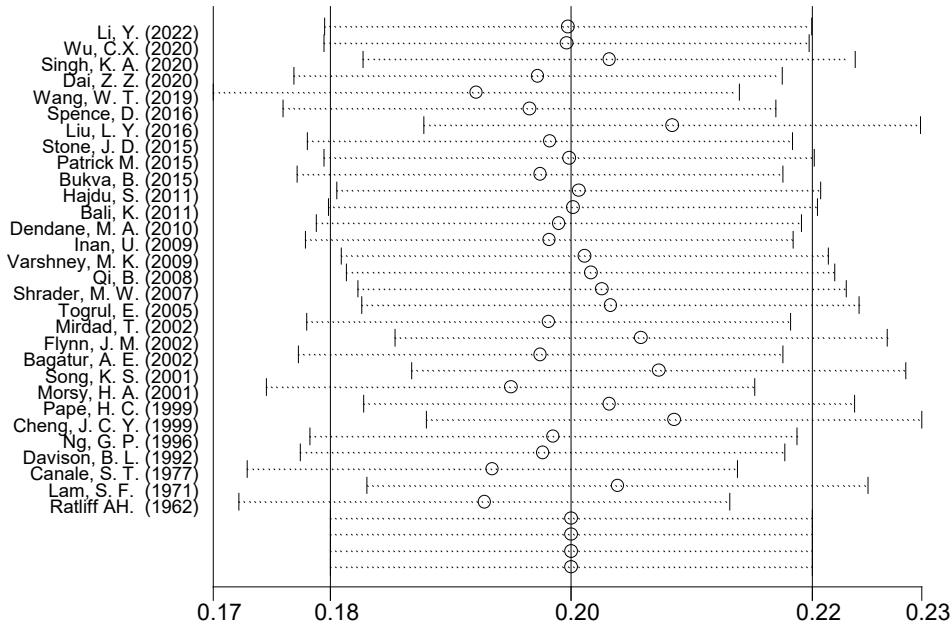

Supplement: Supplementary file 2 — Additional file 2: Sensitivity analysis. [file 13018_2023_3502_MOESM2_ESM.pdf]
